# Autoantibodies against IFNα in patients with systemic lupus erythematosus and susceptibility for infection: a retrospective case-control study

**DOI:** 10.1038/s41598-022-15508-9

**Published:** 2022-07-04

**Authors:** Maxime Beydon, Pascale Nicaise-Roland, Arthur Mageau, Carine Farkh, Eric Daugas, Vincent Descamps, Philippe Dieude, Antoine Dossier, Tiphaine Goulenok, Fatima Farhi, Pierre Mutuon, Jean-Francois Timsit, Thomas Papo, Karim Sacre

**Affiliations:** 1 Service de Médecine Interne, Hôpital Bichat, Assistance Publique Hôpitaux de Paris, 46 rue Henri Huchard, 75018 Paris, France; 2 Service d’Immunologie « Autoimmunité, Hypersensibilités et Biothérapies », Hôpital Bichat, Assistance Publique Hôpitaux de Paris, Paris, France; 3 INSERM UMR1152, Université de Paris, Paris, France; 4 Laboratoire d’Excellence Inflamex, Faculté de Médecine Site Bichat, Centre de Recherche Sur L’Inflammation, INSERM UMR1149, CNRS ERL8252, Université de Paris, Paris, France; 5 IAME UMR1137, Equipe DeScID, Université de Paris, Paris, France; 6 Département de Néphrologie, Hôpital Bichat, Assistance Publique Hôpitaux de Paris, Paris, France; 7 Département de Dermatologie, Hôpital Bichat, Assistance Publique Hôpitaux de Paris, Paris, France; 8 Département de Rhumatologie, Hôpital Bichat, Assistance Publique Hôpitaux de Paris, Paris, France; 9 Département d’Information Médicale, Hôpital Bichat, Assistance Publique Hôpitaux de Paris, Paris, France; 10 Département de Réanimation Médicale Et Infectieuse, Hôpital Bichat, Assistance Publique Hôpitaux de Paris, Paris, France

**Keywords:** Systemic lupus erythematosus, Infectious diseases, Autoimmunity

## Abstract

IFNα and anti-IFNα autoantibodies have been implicated in susceptibility both for systemic lupus erythematosus (SLE) and viral infection. We aimed to analyze the SLE disease phenotype and risk for infection associated with anti-IFN-α IgG autoantibodies in SLE patients In this multidisciplinary retrospective single referral center study, all consecutive patients with SLE admitted between January 1st and November 30th 2020 were considered. All subjects fulfilled the ACR/EULAR 2019 criteria for SLE. Anti-IFNα IgG autoantibodies were quantified at admission by ELISA. Demographic, medical history, laboratory, treatment, and outcome data were extracted from electronic medical records using a standardized data collection form. 180 patients [female 87.2%, median age of 44.4 (34–54.2) years] were included. The median disease duration was 10 years [4–20] with a median SLEDAI score of 2 [0–4] at study time. Fifty-four (30%) patients had a past-history of lupus nephritis. One hundred and forty-four (80%) had received long-term glucocorticoids and 99 (55%) immunosuppressive drugs. Overall, 127 infections—mostly bacterial and viral—were reported in 95 (52.8%) patients. Twenty SLE patients (11.1%) had positive anti-IFNα IgG autoantibodies with a titer ranging from 10 to 103 UA/mL. Age, sex, SLE phenotype and treatment did not significantly differ between SLE patients with or without anti-IFNα. Infection rate was similar in both groups except for tuberculosis which was more frequent in patients with anti-IFNα (20% vs. 3.1%, *p* = 0.01). The prevalence of autoantibodies against IFNα is high in SLE and associated with a higher frequency of tuberculosis.

## Introduction

Type I interferon (mainly IFN-α) has been considered for decades as a pivotal cytokine in SLE^1^. IFN-α, produced mainly by plasmacytoid dendritic cells^2,3^, is known to be associated with disease activity, especially in lupus nephritis^4^ and anti-type I interferon receptor therapy has been shown to be efficient in SLE patients^5^. Recent reports also brought back to light the presence of IFN-α autoantibodies (anti-IFN-α) in up to 40% of SLE patients^6^. Interestingly, anti-IFN-α have been shown to alter the interferon signature and may thus be protective in SLE^7,8^. Anti-IFNα may also predispose to severe COVID-19 by blocking the action of this crucial antiviral cytokine^9^. Thereby, anti-IFN-α in SLE might on one hand help to control the disease and increase the risk of infection on the other hand^10^.

In order to give insight into the complex relationship between infection risk, SLE and anti-IFNα, we analyzed both infection history—including COVID-19—and SLE disease phenotype according to anti-IFN-α status.

## Material and methods

### Population study

All consecutive adult SLE patients admitted between January 1^st^ and November 30^th^ 2020, at Bichat Hospital in 5 distinct Departments of Medicine (Dermatology, Intensive Care Unit, Internal Medicine, Nephrology, and Rheumatology) were selected. Bichat Hospital, Paris, France is a referral national center for rare Autoimmune Diseases. All subjects fulfilled the ACR/EULAR 2019 criteria for Systemic Lupus Erythematosus^11^.

International Classification of Disease code (ICD-10) for SLE (M32) was used for screening. Data were extracted from the French Diagnosis Related Groups (DRG) based information system (PMSI) databases. Demographic, medical history, biological workup, treatment and follow-up data were retrieved from computerized medical files. Exclusion criteria included inability to confirm SLE after review of medical records and inability to retrieve serum specimens for anti-IFN-α analysis. COVID 19 was defined by a positive SARS-CoV-2 RT-PCR testing on a respiratory sample (nasopharyngeal swab or invasive respiratory sample) or a positive serology N-protein specific IgG without prior vaccination. Other prior infections were based on declared history confirmed by medical records.

### Anti-IFNα IgG autoantibodies

The quantification of anti-IFN-α IgG autoantibodies was performed on serum samples draw at admission in the setting of care between January 1^st^ and November 30^th^ 2020. Serum samples were frozen at − 20 °C immediately after collection. Anti-IFN–α IgG were determined by ELISA according to the method described by Bastard et al.^9^. Plates were coated overnight at 4 °C with 1µg/mL recombinant human interferon-α (rhIFN-α2, Miltenyi Biotec) and incubated with 1:50 dilutions of serum samples from the patients. Horseradish peroxidase conjugate Fc specific anti-human IgG (Sigma) was added, the optical density was measured after addition of the substrate (TMB). Arbitrary units were calculated based on a standard curve obtained with the serum of a patient with known high titer of anti-IFNα autoantibodies. The positivity threshold determined in healthy controls was 10 UA/mL.

### Ethical statement

Our study is a human non-interventional retrospective study where 1-study involved products with a marketing authorization that are prescribed in the usual manner and used in accordance with French agencies authorizations, 2-epidemiological methods were used to analyze the data, and 3-information used in the study were collected for clinical care. According to the Public Health French Law (French Research Standard MR-004), approval from institutional review board and written consent are not required for human non-interventional retrospective study. For ethical consideration, patients were however informed that data that was collected in medical records might be used for research study in accordance to privacy rule. The study protocol conforms to the ethical guidelines of the 1975 Declaration of Helsinki.

### Statistical analysis

Continuous variables are expressed as median [IQR]. Categorical variables are expressed as frequencies and percentages. Data were compared between SLE patients with or without anti-IFN-α by using Fisher’s exact test for nominal variables, χ^2^ test for continuous variables with normal distribution and Kruskall-Wallis test for continuous non normal variables. We preplanned 4 analyses of COVID-19, viral infections, zoster, and tuberculosis frequency across groups. A Bonferroni correction taking into account these 4 comparisons was used. Statistical analyses were performed using R software.

## Results

### Characteristics of patients

From January 1st until November 30th 2020, 247 unique SLE patients who had at least an admission in our national referral center (Bichat Hospital, Paris, France) were eligible for the study according to ICD-10 identification. After careful review of individual medical records, the diagnosis of SLE according to ACR/EULAR 2019 criteria was not retained in 67 patients and 180 SLE patients (median age of 44.4 [34–54.2] years, 157 (87.2%) female) were eventually included. Admissions were unplanned (urgent) or planned in 42 (23.3%) and 138 (76.7%) cases, respectively. Unplanned admissions were due to lupus flares (n = 26/42, 61.9%) or infection (n = 7/42, 16.7%) in most cases. Planned admissions were related to SLE follow-up or treatment in 129 (n = 129/138, 93.5%) cases (Fig. S1).

The median SLEDAI disease activity score at admission was 2 [0–4]. Fifty-four (30%) patients had a past history of lupus nephritis. One hundred and forty-four (80%) had received long-term glucocorticoids and 99 (55%) immunosuppressive drugs at some point during follow-up. Steroids were currently prescribed in 62% at a median daily dose of 5 [0–9] mg at study time.

Overall, 127 prior infectious episodes—mainly bacterial (n = 68/127, 53.5%) and viral (n = 40/127, 31.5%)—were reported in 95 (n = 95/180, 52.8%) patients. Viral infection was mostly due to herpes zoster (n = 13/40, 32.5%) and Sars-Cov2 (n = 15/40, 37.5%). Nine (n = 9/180, 5%) patients had tuberculosis (Table 1).

**Table 1:** Characteristic of SLE patients.

	All n = 180	Anti-IFNα positive n = 20	Anti-IFNα negative n = 160	*p*
Female Sex, n (%)	157 (87.2)	17 (85)	140 (87.5)	0.73
Age, years	44.4 [34–54.2]	41.5 [30.25–55]	44.8 [35–54]	0.32
**SLE phenotype**				
Duration of SLE disease, years	10 [4–20]	8.5 [2–20.25]	10 [4–20]	0.58
SLEDAI score	2 [0–4]	2 [0–4.5]	2 [0–4]	0.24
Arthritis, n (%)	127 (71)	13 (65)	114 (71)	0.61
Auto-immune cytopenia, n (%)	37 (21)	1 (5)	36 (22.5)	0.08
Class III/IV nephritis, n (%)	54 (30)	7 (35)	47 (29)	0.61
Serosal, n (%)	49 (27)	5 (25)	44 (28)	1
Neuropsychiatric, n (%)	13 (7.2)	0 (0)	13 (8.1)	0.37
Mucocutaneous, n (%)	125 (69)	14 (70)	111 (69)	1
anti-SSA, n (%)	80 (45)	8 (40)	72 (46)	0.64
anti-SSB, n (%)	22 (12)	1 (5)	21 (13.4)	0.48
anti-RNP, n (%)	51 (29)	8 (40)	43 (27)	0.30
anti-Sm, n (%)	42 (24)	7 (35)	35 (22)	0.26
anti-PL, n (%)	42 (23)	4 (20)	38 (24)	1
Low C3, n (%)	41 (30)	5 (31)	36 (30)	1
Gammaglobulins, g/L	13.8 [10.2–16.8]	11.7 [9.9–17.6]	13.9 [10.75–16.25]	0.9
Lymphocytes count	1.5 [1.1–2]	1.68 [1.1–2]	1.5 [1.1–2]	0.77
GFR < 60 mL/mn/1.73 m^2^, n(%)	12 (7)	2 (10)	10 (6.2)	0.63
Proteinuria/Creatininuria, mg/mmol	30 [20–100]	30 [20–60]	30 [10–100]	0.81
**Infections**				
Bacterium, n (%)	57 (31.7)	4 (20)	53 (33.1)	0.81
Virus	40 (22.2)	6 (30)	34 (21.2)	0.41
VZV	13 (7)	1 (5)	12 (7.5)	1
SarsCov2	15 (8.3)	3 (15)	12 (7.5)	0.22
Admission	7	2	5	–
ICU	0	0	0	–
Asymptomatic	3	1	2	–
Second infection	3	1	2	–
Other$	12 (6.7)	2 (10)	10 (6.2)	0.63
Mycobacterium tuberculosis	9 (5)	4 (20)	5 (3.1)	0.01
Parasite/Fungus, n (%)	10 (5.6)	0 (0)	10 (6.2)	0.61
Pneumocystis carinii	2 (1.1)	0 (0)	2 (1.3)	–
Aspergillosis	0 (0)	0 (0)	0 (0)	–
Other†	8 (4.5)	0 (0)	8 (5)	–
Number of infection				0.72
1	69 (38.3)	9 (45)	60 (37.5)	
2	21 (11.7)	3 (15)	18 (11.2)	
> 2	5 (2.8)	0 (0)	5 (3.1)	
**Treatment history**				
Steroids, n (%)	144 (80)	16 (80)	128 (80)	1
Steroids daily dose*, mg/d	5 [0–9]	5 [0–9.25]	5 [0–9]	0.77
Hydroxychloroquine, n (%)	148 (82)	18 (90)	130 (81)	0.54
HCQ daily dose, mg/d	400 [200–400]	400 [200–400]	400 [375–400]	1
[HCQ] ng/mL	936 [555–1276]	1326 [832–1787]	906 [510–1261]	0.07
Immunosuppressive drugs, n (%)	99 (55)	13 (65)	86 (54)	0.48
Biologics, n (%)	43 (24)	5 (25)	38 (24)	1

*GFR* glomerular filtration rate, *HCQ *hydroxychloroquine, *VZV* varicella-zoster virus, *ICU* intensive care unit, *HCQ* hydroxychloroquine.

Immunosuppressive drugs included cyclophosphamide, azathioprine, mycophenolate mofetil, and methotrexate.

Biologics included belimumab and rituximab.

$other virus include respiratory viruses (n = 2), herpes simplex virus (n = 2), dengue virus (n = 2), hepatitis C virus (n = 2), hepatitis B virus (n = 2), human papillomavirus (n = 1), and chikungunya virus (n = 1).

†Other parasite/fungus include plasmodium falciparum (n = 6), sarcoptes scabiei (n = 1) and cryptosporidium (n = 1).

*Current.

### Anti-IFNα IgG autoantibodies

Twenty SLE patients (11.1%) had positive anti-IFNα IgG autoantibodies with a titer ranging from 10 to 103 UA/mL (median = 15). Anti-IFNα autoantibodies were tested overtime in 54 (30%) patients. When identified at baseline (n = 7), anti-IFNα were confirmed during a follow-up in all but 2 cases. When absent at first screening (n = 47), anti-IFNα remained negative in all but 1 case. Age, sex, SLE features or treatment and history of infection including viral infection rates or types did not differ between SLE patients with or without anti-IFNα, except for tuberculosis disease which was more frequent in patients with anti-IFNα (20% vs 3.1%, *p* = 0.01) (Table 1). In all but 2 cases, TB had occurred a median of 48 [12–168] months before SLE diagnosis in untreated—no immunosuppressive drugs—patients (Table 2).

**Table 2:** SLE patients, anti-IFNα autoantibodies and tuberculosis.

	Anti-IFNα	Age at diagnosis		Gender	SLE Treatment at TB	TB involvement			Other infection
	titer*	TB	SLE			Lung	LN	CNS	
1	< 3	20	42	F	0		1		
2	65	49	53	F	0	1			
3	< 3	35	41	F	0		1		Zona
4	< 3	37	35	F	S, HCQ			1	
5	18	24	24	F	0	1			
6	83	27	27	F	0	1			
7	10	21	45	F	0	1	1		
8	< 3	64	63	F	S, HCQ	1			
9	< 3	52	53	M	0	1	1		

*S* steroids, *HCQ* hydroxychloroquine, *LN* lymph node, *CNS* central nervous system, *F*, female, *M* male, *TB* tuberculosis, *SLE* systemic lupus erythematosus.

*Positivity threshold ≥ 10 UA/mL.

## Discussion

Our study shows that, in a large cohort of carefully characterized SLE patients, 11% are positive for anti-IFNα IgG autoantibodies. This finding is consistent with previous reports on prevalence of anti-INFα antibodies in SLE^7,12^. Our study confirms the high prevalence of anti-IFNα in SLE as compared to the general population where the prevalence is estimated less than 1%^9^.

We found no significant correlation between anti-INFα autoantibodies and SLE disease activity. Published results are conflicting since anti-INFα antibodies have been associated with either increased^7^ or decreased^8^ lupus activity. The fact that almost all SLE patients in our cohort had low disease activity status may have impeded the proper analysis of anti-INFα impact on SLE activity based on SLEDAI score (Fig. S2). In vitro anti-IFNα autoantibodies inhibit IFNα signaling in SLE^7^ and may influence the disease phenotype. In our study, auto-immune cytopenia—mostly immune thrombocytopenia (ITP)—tended to be less frequent in SLE patients immunized against IFNα. Interestingly, IFNα and downstream interferon response genes are known to contribute to the pathogenesis of ITP^13^.

In the general population, neutralizing anti-IFNα IgG autoantibodies are detected in about 10% of patients with severe COVID-19^9^. Anti-IFNα testing may help to identify individuals at high risk of life-threatening infection. Despite a high prevalence of anti-IFNα IgG autoantibodies in SLE and a poor prognosis of severe COVID-19 among patients with SLE^14,15^, no patients displayed severe COVID-19 in our series.

Anti-IFNα autoantibodies were associated with a higher frequency of TB in our SLE patients. Although SLE patients are known to be at higher risk of TB^16^, the interconnection of TB, SLE and anti-IFNα autoantibodies is not easy to decipher. First, since TB preceded SLE in most cases, the implication of SLE treatment is unlikely. Second, TB involved the lungs in 2/3 of cases as reported in the general population^17^. Third, IFNα, in contrast to IFN-γ^18^, is not known to play a key role in susceptibility for TB. Of note, the breach of tolerance observed in SLE triggers a broad range of autoantibodies including anti-IFNα and IFN-γ^7,12^ autoantibodies. In human SLE, large case–control studies have shown that autoantibodies—especially antinuclear antibodies—are present in serum months to years before clinical disease onset^19^. No specific study has addressed the timing of anti-IFNα in the course of SLE. In our study, serial testing over a short period showed that in most SLE patients the anti-IFNα positive or negative status remained stable. Such issues could be addressed prospectively by using serial anti-IFNα testing both in SLE and TB patients.

Our study presents several limitations. First, the study is a retrospective single center study. Second, it has limited power due to the rather limited prevalence of anti-IFNα autoantibodies in SLE even though it is much higher than in the general population. Third, repeated anti-IFNα testing was performed in only a third of patients. Fourth, the neutralizing activity of the autoantibodies on type I IFN was not tested in vitro.

## Conclusion

The prevalence of autoantibodies against IFNα is high in SLE and unexpectedly associated with a high frequency of past tuberculosis.

## Supplementary Information

Supplementary Information.

## Data Availability

All data generated or analysed during this study are included in this published article.

## Abbreviations

ACR: American college of rheumatology
CNS: Central nervous system
COVID: Coronavirus infectious disease
DRG: Diagnosis related groups
EULAR: European alliance of associations for rheumatology
F: Female
GFR: Glomerular filtration rate
HCQ: Hydroxychloroquine
ICU: Intensive care unit
ICD: International classification of disease
IFN: Interferon
ITP: Immune thrombocytopenia
LN: Lymph node
M: Male
PMSI: Programme de médicalisation des systèmes d'information
S: Steroids
SLE: Systemic lupus erythematosus
SLEDAI: Systemic lupus erythematosus disease activity index
TB: Tuberculosis
VZV: Varicella-zoster virus
