# Supplementary figures and images for: Autoantibodies against IFNα in patients with systemic lupus erythematosus and susceptibility for infection: a retrospective case-control study
Source: Sci Rep. 2022 Jul 4;12:11244. doi: 10.1038/s41598-022-15508-9 (PMC9253327; doi:10.1038/s41598-022-15508-9)

**SUPPLEMENTARY MATERIAL**

**Figure S1 Flow chart**


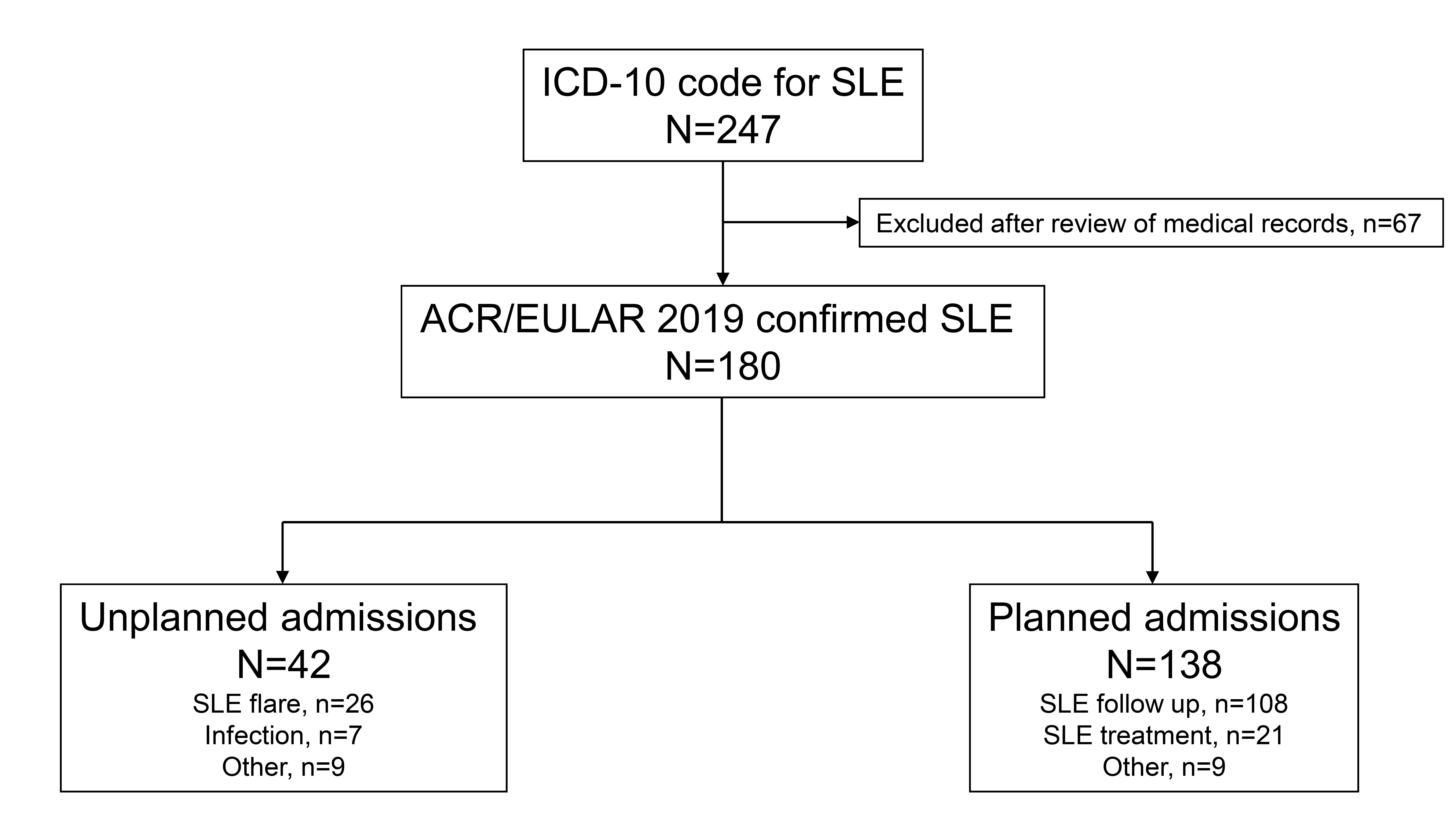


**Figure S2** **Anti-IFNα IgG titers and SLEDAI score**

**
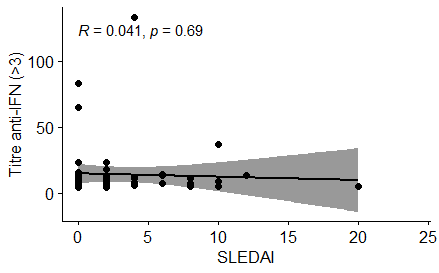
**

Supplement: Supplementary file 1 — Supplementary Information. [file 41598_2022_15508_MOESM1_ESM.docx]
